# *Ophidiomyces ophiodiicola*, Etiologic Agent of Snake Fungal Disease, in Europe since Late 1950s

**DOI:** 10.3201/eid2810.220564

**Published:** 2022-10

**Authors:** Francesco C. Origgi, Simone R.R. Pisano, Olivier Glaizot, Stefan T. Hertwig, Andreas Schmitz, Sylvain Ursenbacher

**Affiliations:** University of Bern, Bern, Switzerland (F.C. Origgi, S.R.R. Pisano, S.T. Hertwig);; Museum of Zoology, Lausanne, Switzerland (O. Glaizot); University of Lausanne, Lausanne (O. Glaizot);; Naturhistorisches Museum Burgergemeinde Bern, Bern (S.T. Hertwig);; Natural History Museum of Geneva, Geneva, Switzerland (A. Schmitz);; University of Basel, Basel, Switzerland (S. Ursenbacher);; info fauna CSCF & karch, Neuchâtel, Switzerland (S. Ursenbacher).

**Keywords:** snake fungal disease, *Ophiodiomyces ophiodiicola*, wildlife, infection, fungi, conservation, reptiles, Switzerland

## Abstract

The fungus *Ophidiomyces ophiodiicola* is the etiologic agent of snake fungal disease. Recent findings date US occurrence at least as far back as 1945. We analyzed 22 free-ranging snakes with gross lesions consistent with snake fungal disease from museum collections from Europe. We found 5 positive samples, the oldest collected in 1959.

In the past few decades, fungal agents have surfaced as relevant threats to conservation and biodiversity among both ectotherms and endotherms (*1*–*3*). The emerging fungal agent *Ophidiomyces ophiodiicola* has been detected in both captive and free-ranging snakes in the United States in the past 16 years (*4*–*7*) and more recently in the United Kingdom and Czech Republic (*8*). *O. ophiodiicola* fungus has been associated with a variably severe dermatitis but also multisystemic disease (*9*). Experimental infections (*10*) demonstrated the causative association between *O. ophiodiicola* infection and snake fungal disease (SFD), the common name attributed to the disease caused by this fungus. The effect on free-ranging populations of snakes is not completely understood, but many species of snakes appear to be susceptible and different populations appear to have been negatively affected (*9*,*11*).

A recent article showed that the earliest evidence of *O. ophiodiicola* infection in North America dates back to 1945 (*12*). However, records of *O. ophiodiicola* fungi in Europe date back only to 2010–2016 (*8*). Detection of 2 phylogenetically distinct lineages in the United States and Europe consistent with genetic differences between the clades, presumably reflects independent evolution of the lineages. To acquire additional data about the origins of this agent in Europe, we obtained skin samples from free-ranging snake collections from multiple natural history museums in Switzerland.

## The Study

We selected 22 skin samples with macroscopic lesions consistent with SFD out of 1,100 free ranging snakes examined from the collections of 3 natural history museums in Switzerland (Table 1; Appendix). We collected tissue samples from the integument of snakes showing obvious macroscopic lesions consistent with dermatitis (Appendix Figure 1). Snake specimens were preserved in 100% ethanol. We collected tissue samples using sterile instrumentation changed between each sampling. We placed each tissue sample in a cryotube containing an aliquot of absolute ethanol. Upon delivery at the laboratory, tissue samples were split into 2 portions for processing for DNA extraction and histopathology (Appendix). 

**Table 1:** Museum tissue samples from snakes of genuses *Natrix* and *Vipera* used in investigation of snake fungal disease in Europe*

Sample	Museum	Species	ID	Sex	Year	Location (country)
**1**	**NMBE**	***Natrix helvetica***	**1049780**	**F**	**2001**	**Erlach (Switzerland)**
2†	NMBE	*N. helvetica*	1056184	NA	2007	Tavannes (Switzerland)
3‡	NMBE	*Vipera aspis*	1072979	NA	2015	Grandvillars (Switzerland)
4	MHNG	*N. tessellata*	1402.040	F	1972	Lake Geneva (Switzerland)
5‡	MHNG	*N. natrix*	851.077	NA	NA	NS (Czech Republic)
**6**	**MHNG**	***N. natrix***	**1342.87**	**NA**	**1963**	**Thurgau (Switzerland)**
**7**	**MHNG**	***N. helvetica***	**1137.18**	**NA**	**1967**	**NS (Italy)**
8†	MHNG	*N. tessellata*	1386.55	F	1969	Tessin (Switzerland)
**9**	**MHNG**	***N. helvetica***	**1397.21**	**NA**	**1959**	**NS (Italy)**
10	MHNG	*N. helvetica*	2430.91	NA	1986	Zurich (Switzerland)
11†	MHNG	*N. maura*	1199.084	F	1971	Haute-Savoie (France)
**12**	**MHNG**	***N. tessellata***	**1387.60**	**F**	**1961**	**Maggia (Switzerland)**
13	MZL	*N. tessellata*	MZL41123	F	2008	Lake Geneva (Switzerland)
14†	MZL	*N. tessellata*	MZL30407	F	2007	Lake Geneva (Switzerland)
15‡	MZL	*N. tessellata*	MZL41142	M	2009	Lake Geneva (Switzerland)
16	MZL	*N. tessellata*	MZL30508	F	2007	Lake Geneva (Switzerland)
17‡	MZL	*N. tessellata*	MZL40905	F	2012	Lake Geneva (Switzerland)
18	MZL	*N. tessellata*	MZL31837	F	2010	Lake Geneva (Switzerland)
19	MZL	*N. tessellata*	MZL30505	NA	2007	Lake Geneva (Switzerland)
20	MZL	*N. tessellata*	MZL41144	F	2009	Lake Geneva (Switzerland)
21†	MZL	*N. tessellata*	MZL40911	F	2013	Lake Geneva (Switzerland)
22	MZL	*N. tessellata*	MZL31839	M	2010	Lake Geneva (Switzerland)

*Bold indicated PCR-positive samples with presence of fungal hyphae. MZL, Museum of Zoology, Lausanne; NMBE, Natural History Museum of Bern; MHNG, Natural History Museum of Geneva; NA, not available; NS, not specified. †PCR-negative samples with presence of fungal hyphae and with histological lesions similar to those observed in the PCR-positive samples. ‡PCR-negative samples with presence of fungal hyphae and with histological lesions dissimilar to those observed in the PCR-positive samples.

We performed PCR according to various protocols aiming to detect multiple gene targets belonging to the *O. ophiodiicola* genome. Initial screening for the presence of *O. ophiodiicola* fungi was performed by applying a modified PCR protocol (an original protocol performed in a conventional PCR setting) (*13*) targeting the partial sequence of the intergenic spacer (IGS). We then tested positive samples and, later, negative samples to rule out false-negative results by IGS PCR by using 3 additional newly developed protocols targeting distinct genome sequences: the 5.8–28s RNA internal transcribed spacer (ITS) 2, the transcription elongation factor (TEF), and the actin genes (Appendix). We used nucleotide sequences obtained from each of the readable PCR amplicons for phylogenetic analysis. We used partial sequences from the amplified ITS, TEF, and actin targets to build up a maximum-likelihood phylogenetic tree for each of the amplified genomic sequences (Appendix). All 22 samples examined for the presence of *O. ophiodiicola* genomic DNA were characterized by gross and microscopic lesions consistent with dermatitis (Table 2; Appendix Figures 1–3).

**Table 2:** Histologic findings from investigation of snake fungal disease in Europe*

Sample	Light microscopy descriptions	PAS findings	Score†
1	Epidermal hyperplasia with serocellular crusts and histiocytic granulomas; mononuclear to heterophilic dermatitis	Septate fungal hyphae, 3 µm thick, branching both at 90 and 45 degrees	3
2	Epidermal hyperplasia with serocellular crusts and microabscesses	Rare, septate fungal hyphae, 2–3 µm thick, branching at 90 degrees	2
3	Epidermal ulceration with heterophilic infiltration and histiocytic dermatitis, intralesional bacteria and foreign material	Septate fungal hyphae, 3 µm thick, branching at 90 degrees	1
4	Ulcerative dermatitis with serocellular crusts and hyperkeratosis	No evidence of fungal hyphae	0
5	Hyperkeratosis	Septate fungal hyphae, embedded in the keratin, 2–3 µm thick, branching at 90 degrees and acute angle	1
6	Hyperkeratosis with histiocytic (granulomatous) dermatitis	Septate fungal hyphae, 3–4 µm thick, branching at acute angle	3
7	Heterophilic granulomas and microabscesses in the epidermis	Rare fungal hyphae, 3 µm thick embedded or associated with the microgranulomas	3
8	Hyperkeratosis with serocellular crusts, epidermal microgranulomas and lymphocytic dermatitis	Septate fungal hyphae, 3 µm thick, branching at 90 degrees and acute angle	2
9	Large crusts surrounded by histiocytic to heterophilic infiltrate and multifocal microgranulomas	Fungal hyphae in the crusts, 2–3 µm thick	3
10	Few crust fragments admixed with bacteria	No detectable fungal hyphae	0
11	Lympho-histiocytic dermatitis with dermal heterophilic granulomas	Rare fragmented hyphae in the heterophilic granulomas	2
12	Serocellular crusts together with large heterophilic granulomas and more diffused histiocytic infiltration; lympho-histiocytic dermatitis	Septate fungal hyphae, 3 µm thick, branching at 90 degrees or acute angle	3
13	Small serocellular crusts	No evidence of fungal hyphae	0
14	Small and rare heterophilic granulomas	Fragments of fungal hyphae in microgranulomas	2
15	A small serocellular crust	Few fungal septate hyphae, 2–3 µm thick, branching at 90 degrees	1
16	Severe dermal edema with isolated inflammatory cells	No obvious fungal elements	0
17	Serocellular crusts with intralesional bacteria	Fragments of non-septate hyphae	1
18	Hyperkeratosis with upper keratin heterophilic to histiocytic infiltration	No obvious fungal elements	0
19	Serocellular crust	No obvious fungal elements	0
20	Intradermal heterophilic granulomas	No obvious fungal elements	0
21	Epidermal heterophilic granulomas with serocellular crusts	Septate fungal hyphae, 2–3 µm thick, branching at 90 degrees	2
22	Intraepidermal crusts with heterophilic granulomas and intralesional bacteria	No obvious fungal elements	0

*PAS, periodic acid–Schiff. †Subjective scoring system complementing morphologic and molecular data; 0, PCR-negative with no histologic evidence of fungi; 1, PCR-negative with presence of fungi but without lesions consistent with those observed in PCR-positive samples (absence of heterophilic granulomas); 2, PCR-negative with presence of fungi and lesions consistent with snake fungal disease; 3, PCR-positive with presence of fungi consistent with *Ophidiomyces ophiodiicola*.

Overall, we observed fungal elements in 14/22 examined tissue sections. All samples positive for SFD by PCR were characterized by the presence of intralesional fungal hyphae and heterophilic granulomas or microabscesses (Appendix Figures 2, 3); we observed intradermal granulomas in 1 sample, in which we could not histologically detect any fungal elements. When we used the original IGS-PCR protocol (*13*), 5/22 samples yielded a detectable band (sample numbers 1, 6, 7, 9, and 12). Samples 6, 7, 9, and 12 were also confirmed positive when we used the ITS primer set. Four of 22 samples (6, 7, 9, and 12) yielded a detectable band when we used the actin primer set. Two of 22 samples (9 and 12) yielded a detectable band when we used the TEF primer set. Despite positive IGS amplification, we could not amplify sample 1 with either the actin or the TEF primer sets. We obtained a nonspecific amplification with the ITS primer set and consequently did not further consider sample 1 for sequence comparison and phylogenetic analysis (Appendix, Figure 4).

Sequence alignments, reflected in the phylogenetic trees (Appendix Figure 4), showed unique single-nucleotide polymorphisms clearly separating the museum samples from Switzerland into either the clade circulating in Europe or the one circulating in North America (Figure) (*8*). Results were consistent across the partial sequences of the targeted ITS, TEF, and actin genomic regions. Specifically, samples 7 and 9 from Italy always clustered within the clade from Europe, whereas 6 and 12 from Switzerland clustered within the clade from North America (Appendix Figure 4). 

**Figure:**
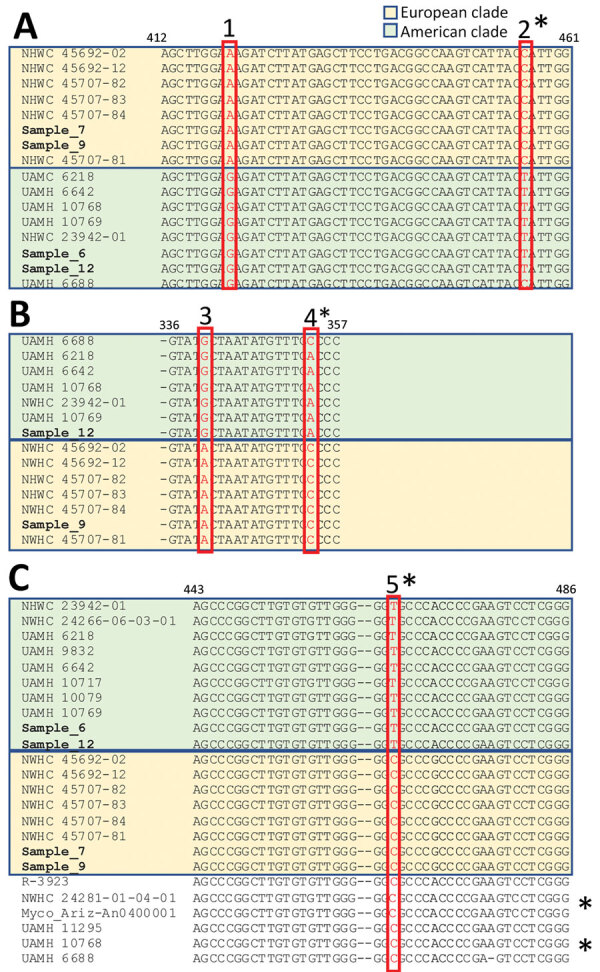
Nucleotide sequence alignment of selected sections of *Ophidiomyces ophiodiicola* from free-ranging snake collections from multiple natural history museums in Switzerland (bold) compared with reference sequences. Amplicons obtained with different PCR primer sets highlight single-nucleotide polymorphisms (SNPs, red boxes) unique to either the European (pastel gold) or American (pastel green) clades. PCR primer results: A) actin; B) transcription elongation factor; and C) internal transcribed spacer. The isolate UAMH 6688 (UK strain) shares 2/5 unique SNPs with the members of the clade from North America, whereas 3 of them (single asterisks) are shared with strains from Europe. These differences match the divergent branching of this strain in the clades from both North America and Europe. Similarly, 5 others fungal isolates (double asterisks)—R-3923; NWHC 24281-01-04-01, Myco_Ariz-An0400001, UAMH 11295, and UAMH 10768, in addition to UAMH 6688, originating from the United States, Australia, and the United Kingdom—shared the internal transcribed spacer SNP of the clade from Europe and clustered consistently in an intermediate group in the corresponding phylogenetic tree (Appendix Figure 4).

## Conclusions

Our research, conducted similarly to an investigation performed in North America, provided evidence of the presence of *O. ophiodiicola* infection in free-ranging snakes in Europe at least since 1959 (*12*). Our findings were supported by test results for 4 distinct molecular targets and consistent histological findings. Furthermore, all PCR-positive samples confirmed by sequencing were also associated with the presence of intralesional fungal structures consistent with *O. ophiodiicola* and associated with an obvious inflammatory reaction.

Of note, supporting data are consistent with the surprising finding that the proposed clades from both North America and Europe (*8*) have been present at least since the early 1960s. Furthermore, because our dataset spanned only 1959–2012, *O. ophiodiicola* fungi might have been present in Europe even before 1959. The significance of both clades existing in Europe will require further investigations. In spite of the absence in the United States of any strain proven to belong to the clade from Europe, introduction cannot be completely ruled out (*14*). In an alternative scenario, the clade from North America might have been introduced into Europe before the 1950s. At the moment, the colonization of *O. ophiodiicola* fungi on the European continent appears to have occurred several decades before proposed (*8*). 

Detection of *O. ophiodiicola* fungi in Italy and Switzerland north of the Alps, further expands its known distribution in Europe. Curiously, Switzerland appears to be the only country in Europe where the clade of *O. ophiodiicola* fungi from North America has to date been identified. However, sampling bias secondary to the restricted sampling area selected cannot be ruled out. Finally, although the 2 samples from Switzerland that clustered with the clade from North America were from different regions, the regions are located relatively close geographically to one another (160 km or ≈100 miles).

In summary, this investigation supports the presence of *O. ophiodiicola* fungi in Europe since at least 1959 with genomic sequences compatible with the 2 known lineages. These results provide critical elements for helping to rethink disease ecology and global distribution of *O. ophiodiicola* fungi and reconstructing its natural history.

Appendix Additional information about study of *Ophiodiomyces ophiodiicola* fungi in snakes in Europe.
