# Supplementary material for: Ophidiomyces ophiodiicola, Etiologic Agent of Snake Fungal Disease, in Europe since Late 1950s
Source: Emerg Infect Dis. 2022 Oct;28(10):2064–8. doi: 10.3201/eid2810.220564 (PMC9514351; doi:10.3201/eid2810.220564)
Supplement: Appendix — Additional information about study of Ophiodiomyces ophiodiicola fungi in snakes in Europe. [file 22-0564-Techapp-s1.pdf]

Article DOI: https://doi.org/10.3201/eid2810.220564

Ophidiomyces ophiodiicola, Etiologic Agent of Snake Fungal Disease, in Europe since Late 1950s

Appendix

Additional Methodology

Sample Selection

We assessed 1,100 individual snakes, mainly *Natrix* spp., *Hierophis viridiflavus*, and a few vipers (*V. berus*). Most (≈95%) were from Switzerland; the remaining snakes were from the surrounding countries, mainly Germany and Italy, and a few from France and Czech Republic. While they were still in preservation jars, we visually assessed these snake specimens by examining the dorsal and ventral scales; when putative dermatitis was observed (macroscopic darkening of the scales with pitting, fragmentation, lifting, and detachment), we removed the snakes from the jars and observed them more closely. If a lesion was confirmed on closer inspection, then we sampled the snake. We included only adult and subadult snakes in this study.

Tissue Processing for DNA Extraction and Histological Slide Preparation

DNA extraction was performed using the Qiagen DNeasy extraction kit (https://www.qiagen.com) according to the manufacturer's instruction. DNA amount and purity was assessed using the ThermoFisher Nanodrop spectrophotometer (https://www.thermofisher.com) and stored at 4°C prior downstream analysis. Histopathology was performed following the current protocols used at the Institute of Animal Pathology (ITPA) of the University of Bern. Briefly, the tissues were first dehydrated and then embedded in paraffin blocks, which were then sectioned 5 μm thick and stained with hematoxylin and eosin. Periodic acid Schiff (PAS), a special stain commonly used to detect fungal organisms in tissues, was applied to each of the examined sections.

Molecular Investigation

Given that the tissues samples from the museums had been fixed for up to several decades and DNA fragmentation might have occurred, we designed specific primers

amplifying DNA fragments no longer than 200bp. More specifically a forward (5′-TGTCCGAGCGTCATTGCAACC-3′) and reverse (5′-AACAGATTCCCATACACTCAGACACC-3′) primer amplifying the partial sequence of the ITS, a forward (5′-CCAGCCCAACTATCAAACTTTGGC-3′) and a reverse (5′-TGATACCACGCTCACGCTCGG-3′) primer amplifying the partial sequence of the TEF gene, and a forward (5′-TTAGATTTCCAGCAAGAGATCCAGACTG-3′) and a reverse (5′-CCAAGACGCTGGGTTGGAAAAG-3′) primer amplifying the partial sequence of the ACT gene were designed. The primer sets were selected attempting to target the most polymorphic regions across the selected sequences. DNA amplification was carried similarly for all the different targets. More specifically, 1 μL of 100 μM suspension of the selected forward and reverse primer were added to a mix including 3.75 μL of a 25 mM $MgCl_2$ solution, 3 μL of 10X buffer, 0.4 μL of a 10 mM dNTPs mix, 0.2 μL Firepol DNA polymerase (Solis BioDyne-Lucerna-chem, CH), 100 ng of DNA target, and ddH_20 up to 30 μL. Cycling included a denaturing step at 94°C for 3 minutes followed by 40 cycles comprising a 30 second long 94°C denaturing step, an annealing step at 52°C for 30 seconds and an extension step at 72°C for 30 seconds. A 10 minutes long final extension step at 72°C followed. The samples were then resolved in a 2% agarose gel and examined under UV lights.

Phylogenic Analysis

The phylogenetic trees were built by comparing the sequences obtained from the investigated samples in this study together with a set of homologous sequences available in GenBank comprising strains belonging either to the European or the North American lineage of *O. ophiodiicola* together with an unrelated fungus serving as outgroup (*Pseudoamauroascus australiensis*) (**ACT**: KY474070.1 = NWHC 45692–02; KY474071.1 = NWHC 45692–12; KY474073.1 = NWHC 45707–82; KY474074.1 = NWHC 45707–83; KY474075.1 = NWHC 45707–84; KY474072.1 = NWHC 45707–81; KY474066.1 = UAMH 6218; KY474067.1 = UAMH 6642; KY474068.1 = UAMH 10768; KY474069.1 = UAMH 10769; KY474076.1 = NWHC 23942–01; KY474078.1 = UAMH 6688; UAMH8392 = *Pseudoamauroascus australiensis* outgroup; **TEF**: KY474092.1 = *Pseudoamauroascus australiensis* (UAMH 8392) outgroup; KY474082.1 = UAMH 6688; KY474080.1 = UAMH 6218; KY474081.1 = UAMH 6642; KY474083.1 = UAMH 10768; KY474091.1 = NWHC 23942–01; KY474084.1 = UAMH 10769; KY474085.1 = NWHC 45692–02; KY474086.1 = NWHC 45692–12; KY474088.1 = NWHC 45707–82; KY474089.1 = NWHC 45707–83; KY474090.1 = NWHC

45707–84; KY474087.1 = NWHC 45707–81; **ITS**: AJ131787.1 = *Pseudoamauroascus australiensis* outgroup; KY474065.1 = NWHC 23942–01; KX148657.1 = NWHC 24266–06–03–01; KF477227.1 = UAMH 6218; KF477229.1 = UAMH 9832; KC884267.1 = UAMH 6642; KF477233.1 = UAMH 10717; KF477231.1 = UAMH 10079; KF477235.1 = UAMH 10769; KY474059.1 = NWHC 45692–02; KY474060.1 = NWHC 45692–12; KY474062.1 = NWHC 45707–82; KY474063.1 = NWHC 45707–83; KY474064.1 = NWHC 45707–84; KY474061.1 = NWHC 45707–81; EU715819.1 = R-3923; KX148658.1 = NWHC 24281–01–04–01; KF225599.1 = MYCO-ARIZ AN0400001; KF477237.1 = UAMH 11295; KF477234.1 = UAMH 10768).

The nucleotide sequences were aligned using Muscle (https://www.ebi.ac.uk/Tools/msa/muscle) with standard settings and the resulting alignments were fed into Mega 7 (MEGA7: Molecular Evolutionary Genetics Analysis version 7.0 for bigger datasets. Kumar S, Stecher G, Tamura K. MEGA7: Molecular Evolutionary Genetics Analysis Version 7.0 for Bigger Datasets. Mol Biol Evol. 2016;33:1870–4.) to obtain a maximum likelihood phylogenetic tree. The substitution model used was the Tamura-Nei model with uniform rates among sites, complete deletion of gaps, 10,000 bootstrap replications. The alignments were 93 nt long for the ACT, 394 nt for the TEF, and 272 nt for the ITS trees.

Sequences

Here are listed all the sequences obtained during this investigation. For each sequence, length of the amplicon (with excised primers) and nucleotide mismatches are reported, shown bolded.

IGS (partial sequence of the intergenic spacer**)** 97 nt without primers

>Sample_1
TTACATTTTCCATACAAAAAGGTGG———G TCCGGCGACCC**C**GGGAAAACCCTCG-
TTCCGCGGAAGACAAGCGCCCGGAGAGTAT

>Sample_6
TTACATTTTCCATACAAAAAGGTGGTCCCGACCCAGTCAGGTCCGGCGACCC**A**GGGAAAACCCTCGGATCCGCGG
AAGACAAGCGCCCGGAGAGTAT

>Sample_7
TTACATTTTCCATACAAAAAGGTGGTCCCGACCCAGTCAGGTCCGGCGACCC**C**GGGAAAACCCTCGGATCCGCGG
AAGACAAGCGCCCGGAGAGTAT

>Sample_9
TTACATTTTCCATACAAAAAGGTGGTCCCGACCCAGTCAGGTCCGGCGACCC**C**GGGAAAACCCTCGGATCCGCGG
AAGACAAGCGCCCGGAGAGTAT

>Sample_12
TTACATTTTCCATACAAAAAGGTGGTCCCGACCCAGTCAGGTCCGGCGACCC**A**GGGAAAACCCTCGGATCCGCGG
AAGACAAGCGCCCGGAGAGTAT

ITS (5.8–28s RNA internal transcribed spacer 2**)** 100 nt without primers

>Sample_1 (non-specific amplification; not included in phylogenetic analysis)
___GTCCGAGTTGTCCGAGCGTCATTGCAACCCTGGACCATGCAGGTTGCTAAATGAGAAGAAGGTAgAGTCCCA
G___ TGTNTTAGTGCTGTGGGGTGTCTGAGTGTATGGGAATCTGTTATCTG
>Sample_6
CCCTCAAGCCCGGCTTGTGTGTTGGGGG**T**GCCCACCCCGAAGTCCTCGGGCGCGGG**C**CCCCCCCAAATGCAGTGG
CGGCACCGAGTTCCT
>Sample_7
CCCTCAAGCCCGGCTTGTGTGTTGGGGG**C**GCCCGCCCCGAAGTCCTCGGGCGCGGG-
CCCCCCCAAATGCAGTGGCGGCACCGAGTTCCT
>Sample_9
CCCTCAAGCCCGGCTTGTGTGTTGGGGG**C**GCCCGCCCCGAAGTCCTCGGGCGCGGG-
CCCCCCCAAATGCAGTGGCGGCACCGAGTTCCT
>Sample_12
CCCTCAAGCCCGGCTTGTGTGTTGGGGG**T**GCCCACCCCGAAGTCCTCGGGCGCGGG**C**CCCCCCCAAATGCAGTGG
CGGCACCGAGTTCCT

ACT (actin gene) 92 nt without primers

>Sample_7
CTGCTCAGAGCTCTAGCTTGGA**A**AGATCTTATGAGCTTCCTGACGGCCAAGTCATTAC**C**ATTGGCAACGAGCGAT
TCCGTGCTCCCGAAGCC
>Sample_9
CTGCTCAGAGCTCTAGCTTGGA**A**AGATCTTATGAGCTTCCTGACGGCCAAGTCATTAC**C**ATTGGCAACGAGCGAT
TCCGTGCTCCCGAAGCC
>Sample_6
CTGCTCAGAGCTCTAGCTTGGA**G**AGATCTTATGAGCTTCCTGACGGCCAAGTCATTAC**T**ATTGGCAACGAGCGAT
TCCGTGCTCCCGAAGCC
>Sample_12
CTGCTCAGAGCTCTAGCTTGGA**G**AGATCTTATGAGCTTCCTGACGGCCAAGTCATTAC**T**ATTGGCAACGAGCGAT
TCCGTGCTCCCGAAGCC

TEF (transcription elongation factor) 158 nt without primers

>Sample_9

AGAATTGTCGATCTTTGACCAATCATGCCTGACCCCTTTGAACCATGCATTTTTTACCTTGACGCTCTTCAGTAT
ACTAATATGTTTC**C**CCCCTTAGGAAGCCGAAGAGTTGGGCAAGAAATCCTTCAAATATGCCTGGGTTCTTGACAA
ATTGAAGG
>Sample_12
AGAATTGTCGATCTTTGACCAATCATGCCTGACCCCTTTGAACCATGCATTTTTTACCTTGACGCTCTTCAGTAT
GCTAATATGTTTC**A**CCCCTTAGGAAGCCGAAGAGTTGGGCAAGAAATCCTTCAAATATGCCTGGGTTCTTGACAA
ATTGAAGG

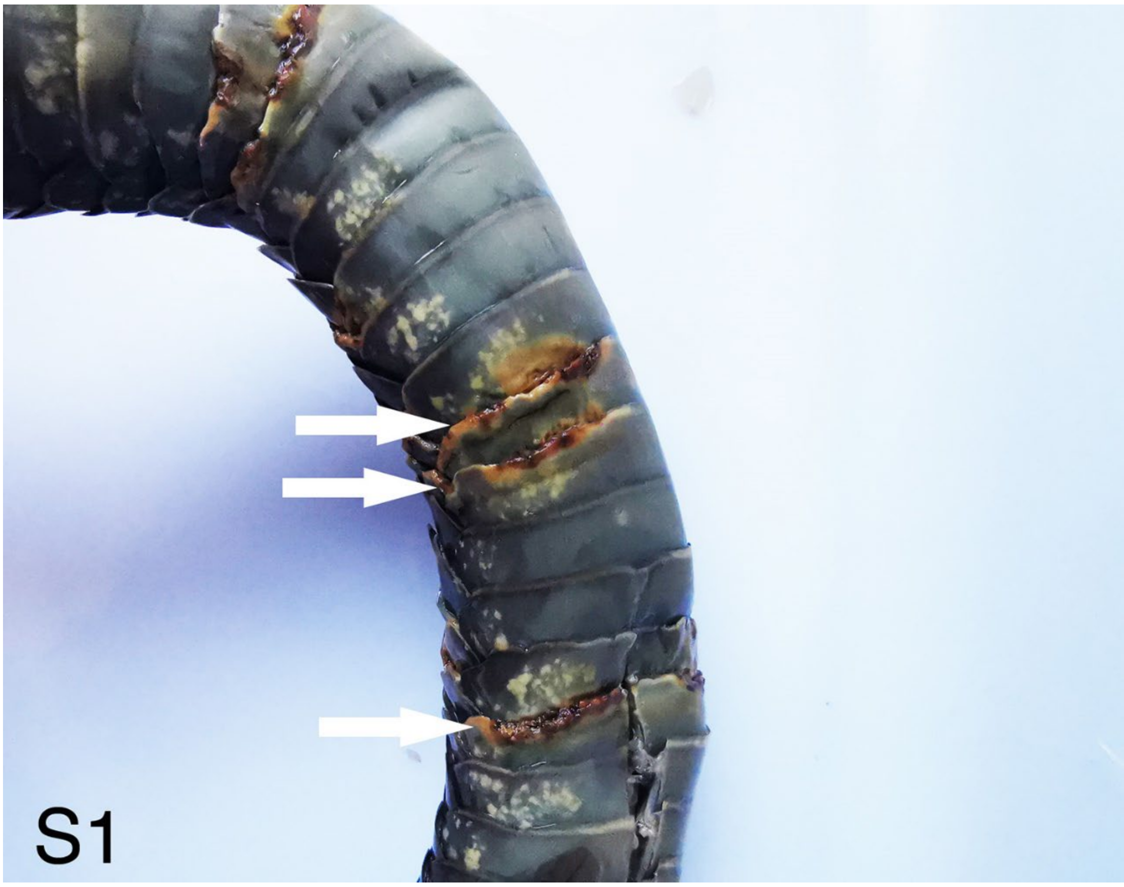

Appendix Figure 1. Skin from *Natrix helvetica* (MHNG1137.18) from study of *Ophidiomyces ophiodiicola*, etiologic agent of snake fungal disease. The caudal rim of several ventral scales is colored dark tan to brown with variably extensive indentation (ulcerative necrosis, white arrows).

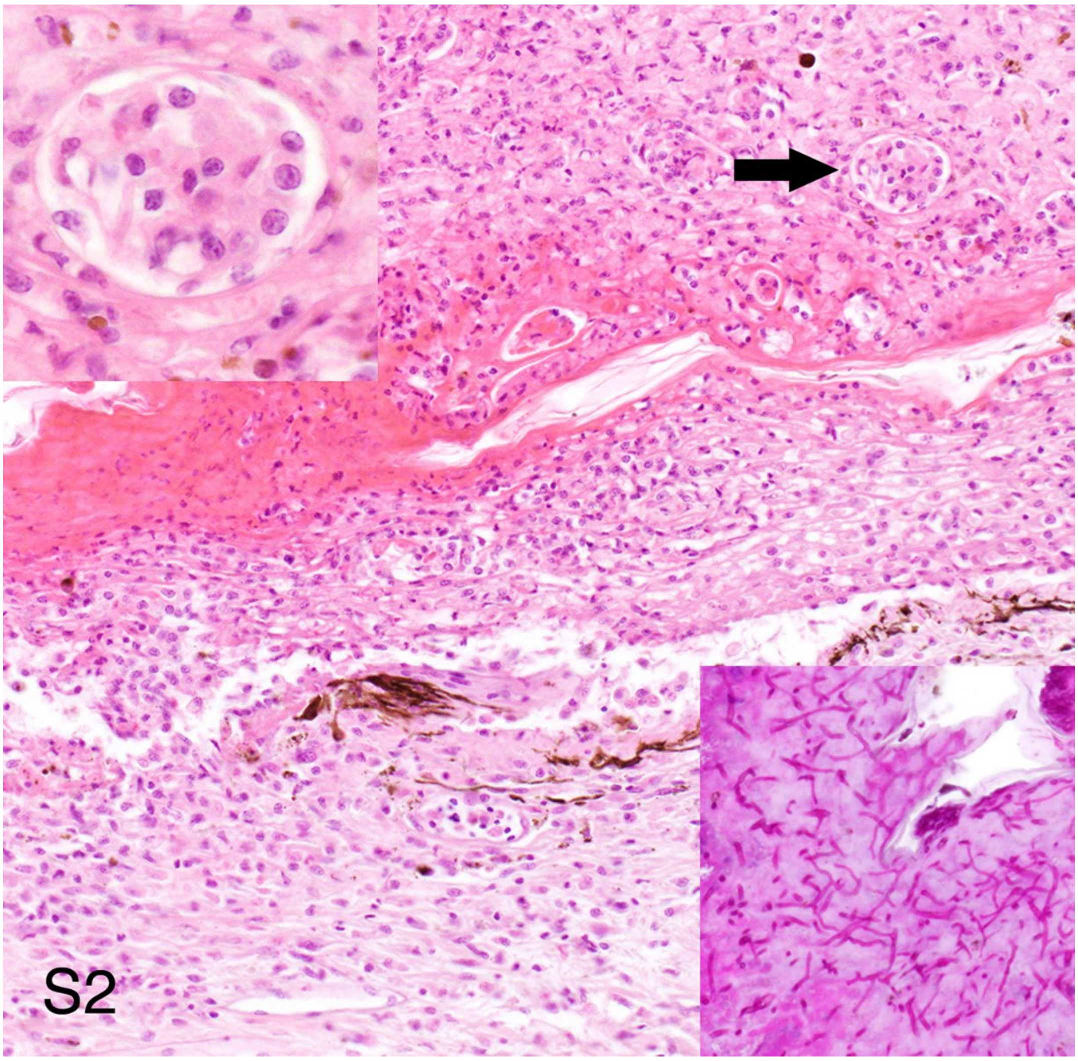

Appendix Figure 2. Skin from *Natrix* sp. from study of *Ophidiomyces ophiodiicola*, etiologic agent of snake fungal disease. The epidermis is diffusely and severely infiltrated by inflammatory cells. Multifocally, organized cores of histiocytic cells (granulomas) are scattered within the effaced epidermis (black arrow and upper left inset). Numerous fungal elements are highlighted by the Periodic acid Schiff (PAS) stain (lower right inset).

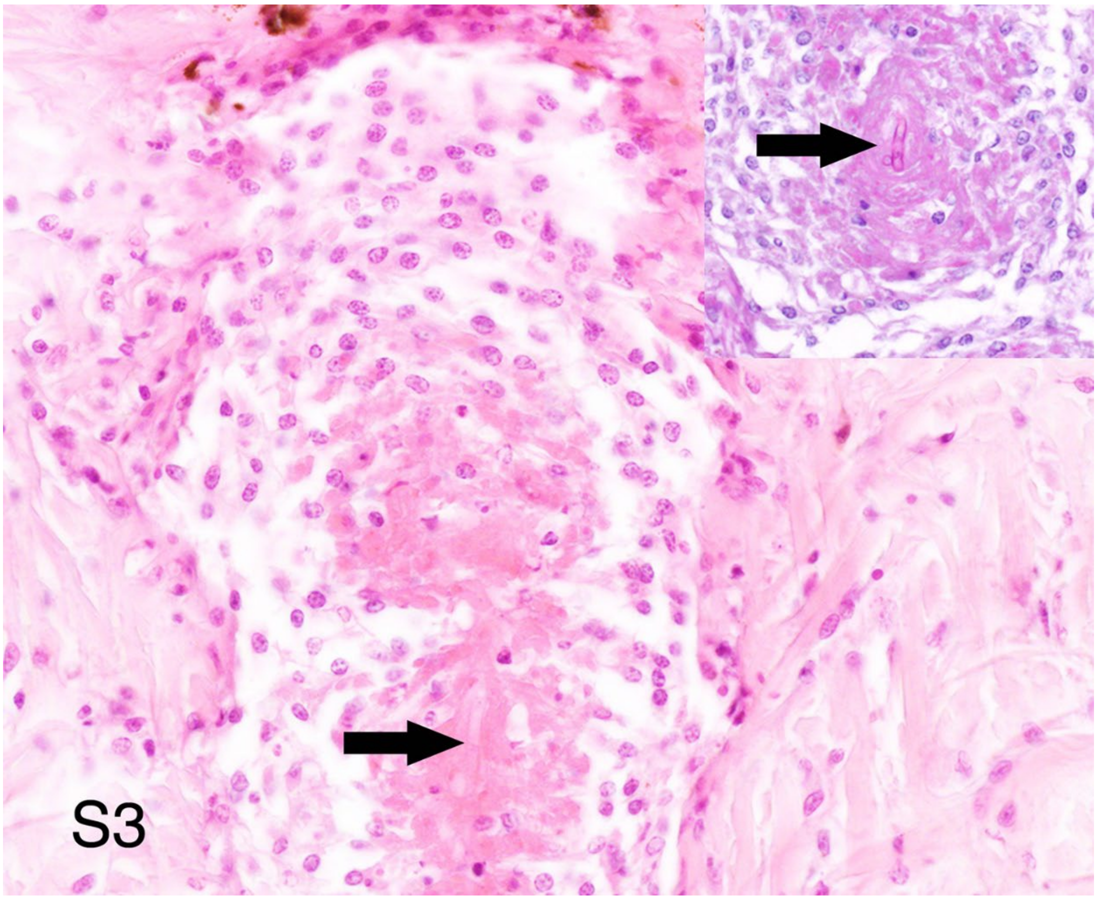

Appendix Figure 3. Skin from *Natrix* sp. from study of *Ophidiomyces ophiodiicola*, etiologic agent of snake fungal disease. A granuloma embedding a fungal hypha (negative image, black arrow [H&E]; black arrow and inset [PAS]) is expanding within the dermis.

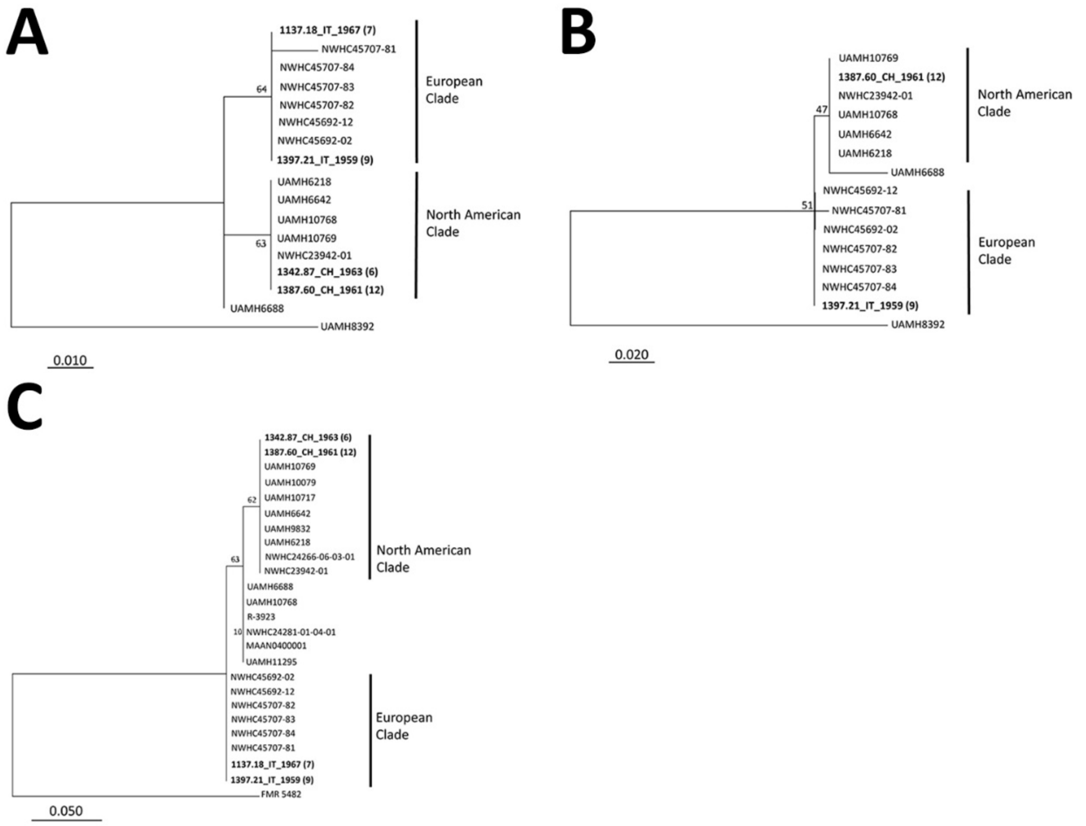

Appendix Figure 4. Phylogenetic analysis of *Ophidiomyces ophiodiicola* clades from North America and Europe, using various PCR primers. Maximum likelihood phylogenetic trees obtained for the A) ACT and B) TEF genes, and C) the 5.8-28s RNA internal transcribed spacer 2. All trees show a clear separation of the clades of *O. ophiodiicola* from Europe and North America. Numbers at the nodes show the bootstrap values. The outgroup is the same fungal species for each tree. The *O. ophiodiicola*–positive samples identified in this study are listed in bold in the trees. The sample number is shown in brackets. ACT, actin; TEF, transcription elongation factor; ITS, internal transcribed spacer
